# The upregulation and transcriptional regulatory mechanisms of Extra spindle pole bodies like 1 in bladder cancer: An immunohistochemistry and high-throughput screening Evaluation

**DOI:** 10.1016/j.heliyon.2024.e31192

**Published:** 2024-05-15

**Authors:** Wei Zhang, Zi-Qian Liang, Rong-Quan He, Zhi-Guang Huang, Xiao-Min Wang, Mao-Yan Wei, Hui-Ling Su, Zhi-Su Liu, Yi-Sheng Zheng, Wan-Ying Huang, Han-Jie Zhang, Yi-Wu Dang, Sheng-Hua Li, Ji-Wen Cheng, Gang Chen, Juan He

**Affiliations:** a Department of Pathology, The First Affiliated Hospital of Guangxi Medical University, 6 Shuangyong RD, Nanning, Guangxi Zhuang Autonomous Region, 530021, PR China; b Department of Medical Oncology, The First Affiliated Hospital of Guangxi Medical University, 6 Shuangyong RD, Nanning, Guangxi Zhuang Autonomous Region, 530021, PR China; c Department of Urology, The First Affiliated Hospital of Guangxi Medical University, 6 Shuangyong RD, Nanning, Guangxi Zhuang Autonomous Region, 530021, PR China

**Keywords:** Gene expression, Epigenetic regulation, ESPL1, miR-299-5p, Early screening

## Abstract

**Background:**

This study aimed to explore the expression level and transcriptional regulation mechanism of Extra Spindle Pole Bodies Like 1 (ESPL1) in bladder cancer (BC).

**Methods:**

A multicentre database of samples (n = 1391) was assayed for ESPL1 mRNA expression in BC and validated at the protein level by immunohistochemical (IHC) staining of in-house samples (n = 202). Single-cell sequencing (scRNA-seq) analysis and enrichment analysis explored ESPL1 distribution and their accompanying molecular mechanisms. ATAC-seq, ChIP-seq and Hi-C data from multiple platforms were used to investigate ESPL1 upstream transcription factors (TFs) and potential epigenetic regulatory mechanisms. Immune-related analysis, drug sensitivity and molecular docking of ESPL1 were also calculated. Furthermore, upstream microRNAs and the binding sites of ESPL1 were predicted. The expression level and early screening efficacy of miR-299-5p in blood (n = 6625) and tissues (n = 537) were examined.

**Results:**

ESPL1 was significantly overexpressed at the mRNA level (p < 0.05, SMD = 0.75; 95 % CI = 0.09, 1.40), and IHC staining of in-house samples verified this finding (*p* < 0.0001). ESPL1 was predominantly distributed in BC epithelial cells. Coexpressed genes of ESPL1 were enriched in cell cycle–related signalling pathways, and ESPL1 might be involved in the communication between epithelial and residual cells in the Hippo, ErbB, PI3K-Akt and Ras signalling pathways. Three TFs (H2AZ, IRF5 and HIF1A) were detected upstream of ESPL1 and presence of promoter-super enhancer and promoter-typical enhancer loops. ESPL1 expression was correlated with various immune cell infiltration levels. ESPL1 expression might promote BC growth and affect the sensitivity and therapeutic efficacy of paclitaxel and gemcitabine in BC patients. As an upstream regulator of ESPL1, miR-299-5p expression was downregulated in both the blood and tissues, possessing great potential for early screening.

**Conclusions:**

ESPL1 expression was upregulated in BC and was mainly distributed in epithelial cells. Elevated ESPL1 expression was associated with TFs at the upstream transcription start site (TSS) and distant chromatin loops of regulatory elements. ESPL1 might be an immune-related predictive and diagnostic marker for BC, and the overexpression of ESPL1 played a cancer-promoting role and affected BC patients’ sensitivity to drug therapy. miR-299-5p was downregulated in BC blood and tissues and was also expected to be a novel marker for early screening.

## 1 Introduction

Bladder cancer (BC) is mainly caused by smoking and occupational exposure to carcinogens and is the most common malignant tumour of the urinary tract [1]. An authoritative survey indicated that, in 2020, there would be approximately 573,000 new cases of BC and 213,000 BC-related deaths worldwide [2]. Despite the efficacy of first-line treatment for BC through the use of gemcitabine and cisplatin, its high level of heterogeneity [3], invasiveness [4] and frequency of recurrence [5,6] continue to be significant challenges, and the five-year survival rate of patients with middle and advanced BC is only 39 % [7,8]. In recent years, it has been reported that histone modifications and noncoding RNAs play essential roles in the transcriptional regulation of genes and that they may serve as early markers for BC [9]. Therefore, the development of early screening and the identification of therapeutic targets for BC treatment warrant further investigation.

Extra spindle pole bodies like 1 (ESPL1) are cysteine endopeptidases, and these proteins participate in several aspects of the cell cycle by activating segregase [10]. There is growing evidence that high expression of ESPL1 is strongly associated with the development of malignant tumours, such as hepatocellular carcinoma (HCC) [11], glioma [12], lung adenocarcinoma (LUAD) [13] and colorectal cancer (CRC) [14]. In addition, Chen et al. found that the high expression of ESPL1 was associated with the occurrence and recurrence of BC [15]. However, their study had some limitations: It included only a small number of samples (n = 246), in-house samples were not included for experimental validation, and there was neither an in-depth exploration of the potential regulatory mechanisms of ESPL1 expression nor a comprehensive analysis at the cellular level. Furthermore, epigenetic modifications and microRNAs (miRNAs) could potentially influence tumour suppression and growth by affecting gene expression; however, the roles that these factors potentially play in the upstream regulation of ESPL1 have not been clarified [16]. Therefore, it is essential to determine the expression and potential epigenetic regulatory mechanisms of ESPL1 in BC.

In the present study, we utilised immunohistochemistry (IHC) to examine ESPL1 expression in in-house and multicentre BC samples at the messenger RNA (mRNA) and protein levels. We also explored the correlations between ESPL1 expression in BC and signalling pathways, immune cell infiltration and clinical value. In addition, we obtained single-cell sequencing (scRNA-seq) data and data on the epigenetic regulation of histone modifications, which provided a novel basis for exploring the expression mechanism, cellular communication and upstream epigenetic regulation of ESPL1. Finally, we predicted the upstream miRNA regulators of ESPL1, discovering the differential expression and early screening efficacy of miR-299-5p in BC.

## 2 Materials and methods

### 2.1 Data collection and processing

The BC-associated mRNA and miRNA samples used in the present study were obtained from the Gene Expression Omnibus (GEO), ArrayExpress and the Cancer Genome Atlas (TCGA). The study included datasets with blood or tissue samples from BC patients with at least three pairs of samples and excluded cohorts with unrecognisable data or who had undergone treatment (e.g.*,* radiation) (Fig. S1). A total of eight miRNA-associated datasets (three blood cohorts and five tissue cohorts) and 19 mRNA tissue datasets were ultimately included (Table 1). The clinical characteristics of the patients can be downloaded directly from the TCGA's website (https://portal.gdc.cancer.gov/).

**Table 1:** Basic information about the high-throughput sequencing datasets included in this study.

Category	Dataset	Platform	Country	Year	BC samples	Non-BC samples
tissue mRNA						
	E-MTAB-1940	–	France	2015	82	4
	GSE65635	GPL14951	Russia	2015	8	4
	GSE86411	GPL14951	USA	2016	132	0
	GSE7476	GPL570	Spain	2007	9	3
	GSE31684	GPL570	USA	2012	93	0
	GSE2109	GPL570	USA	2005	15	8
	GSE31189	GPL570	USA	2013	52	40
	GSE19423	GPL6102	South Korea	2010	48	0
	GSE37815	GPL6102	South Korea	2013	18	6
	GSE13507	GPL6102	South Korea	2010	165	68
	GSE2361	GPL96	USA	2005	0	1
	GSE3167	GPL96	Denmark	2005	41	9
	GSE5287	GPL96	Denmark	2007	30	0
	GSE236932	GPL24676	China	2023	38	25
	GSE24152	GPL6791	USA	2010	10	7
	GSE40355	GPL8227	Germany	2013	16	8
	GSE52519	GPL6884	Russia	2013	9	3
	GSE76211	GPL17586	China	2017	3	3
	TCGA_BLCA_mRNA	–	–	–	414	19
blood miRNA						
	GSE112264	GPL21263	Japan	2019	50	41
	GSE113486	GPL21263	Japan	2018	392	100
	GSE211692	GPL21263	Japan	2022	399	5643
tissue miRNA						
	E-MTAB-2573	–	China	2014	4	4
	GSE236933	GPL24676	China	2023	38	25
	GSE2564-GPL1986	GPL1986	USA	2005	7	2
	GSE39093	GPL8786	China	2012	10	10
	TCGA_BLCA_miRNA	–	–	–	418	19

Note: BC, bladder cancer.

The miRNA and mRNA datasets were processed with log2 (x + 0.01) and log2 (x + 1), respectively, and the ‘limma’ package [17] was used for normalisation. The 19 mRNA datasets were merged to form 11 new cohorts based on the same experimental platform. Immediately thereafter, the batch effect was eliminated using the ‘SVA’ package [18].

The Depmap Portal is a clustered regularly interspaced short palindromic repeats (CRISPR) database for tumour cell lines that has been often used to study the potential dependence of multiple genes in cancer cells [19]. In the present study, the ESPL1 Chronos score dataset (n of samples = 1095), which is associated with CRISPR screens of multiple cancer cell lines, was obtained from the Depmap Portal, and the scores were processed using the CERES algorithm. In addition, we obtained the IHC staining results of BC and normal tissue samples from the Human Protein Atlas (THPA, https://www.proteinatlas.org/) website (BC patient IDs 4947 and 4937).

### 2.2 IHC staining of in-house samples to assess ESPL1 protein levels in BC tissues

To further assess the expression of ESPL1 in BC at the protein level, we collected 202 tissue samples (175 BC and 27 control samples) from the First Affiliated Hospital of Guangxi Medical University and divided them into three tissue microarrays (No. BLC242, BLC1021 and BLC1501; produced by Guilin Fanpu Bio-technology Company, Guangxi Zhuang Autonomous Region, China) for IHC staining. The tissue sections were first fixed in paraffin and then subjected to the sequential addition of xylene for dewaxing and rehydration. The tissue sections were immersed in ethylenediaminetetraacetic acid buffer for antigen repair. Immediately following incubation with hydrogen peroxide (to reduce endogenous peroxidase activity), the sections were treated with anti-ESPL1 rabbit polyclonal antibody (Biorbyt, orb393048; dilution ratio 1:100). The tissue microarrays were then restained, dehydrated, made transparent and sealed at room temperature, with washing with phosphate-buffered saline between steps. The sections were graded according to the level of staining, as follows: negative (no staining) = 0, weakly positive (pale yellow) = 1, positive (brownish yellow) = 2 and strongly positive (brownish black) = 3. The percentage of stained cells in each section was categorised as <5 %, 5–25 %, 26–50 %, 51–75 % or >75 % and the sections were scored 1–4, respectively. Two pathologists individually assessed the total IHC score for each section (the product of the staining score and percentage of positively stained cells), and the resultant original data are shown in Supplementary Material 1.

The present involved human subjects and was approved by the Ethics Committee of the First Affiliated Hospital of Guangxi Medical University (2022-KT-GUOJI-146). All patients who provided in-house samples signed an informed consent form.

### 2.3 Pathway enrichment analysis

Pearson's correlation coefficient *r* was utilised to calculate the coexpressed genes (CEGs) associated with ESPL1 in BC. Genes that met the following criteria were considered to be CEGs of ESPL1: *r* ≥ 0.6 and *p* < 0.05 (positive correlation) and *r* ≤ 0.6 and *p* < 0.05 (negative correlation). When a particular CEG appeared in at least three datasets, it was included in the next analytical step. Positive- and negative-correlated CEGs were analysed separately for pathway enrichment. The ‘clusterProfiler’ package [20] was used for the Kyoto Encyclopedia of Genes and Genomes (KEGG) and Gene Ontology (GO) enrichment analysis, and the ‘ReactomePA’ package [21] was used for Reactome enrichment analysis.

### 2.4 Immune-related analysis

Based on the TCGA_BLCA_mRNA dataset, the ‘IOBR’ package [22] was used for immune-related analysis. The ESTIMATE algorithm was used to study the level of infiltration of tumour stromal cells and immune cells. Furthermore, the CIBERSORT algorithm was used to calculate the scores of 22 immune cells between the ESPL1 high- and low-expression groups. The TIMER algorithm was used to calculate the infiltration level of six immune cells using data extracted from the TIMER database (https://cistrome.shinyapps.io/timer/).

### 2.5 scRNA-seq data processing and comprehensive analysis

In the current study, the GEO GSE176249 dataset was subjected to scRNA-seq. At least three cells expressing ESPL1 and at least 200 genes per cell were selected using the ‘Seurat’ package [23]. The ratio of ribosomal RNA to mitochondria was calculated to select cells with more than 500 expressed genes and less than 25 % mitochondria, which was followed by principal component analysis downscaling. Dim was set at 15 and resolution at 0.5 for the clustering analysis and the uniform manifold approximation and projection (UMAP) algorithm for secondary dimensionality reduction. Finally, the ‘SingleR’ package [24] was used to annotate cell types. The ‘CytoTRACE’ package [25] was used to perform a cellular temporal analysis. In addition, the ‘CellChat’ [26] and ‘CommPath’ [27] packages were utilised to explore cell–cell signalling.

### 2.6 Epigenetic regulatory mechanisms of ESPL1 expression

To further examine the expression of ESPL1 in BC, we undertook an in-depth analysis of its epigenetic regulatory mechanism. Based on the Cistrome Data Browser (http://cistrome.org/db/#/), we predicted the transcription factors (TFs) that bind to the upstream transcription start site (TSS) of ESPL1 and used chromatin immunoprecipitation sequencing (ChIP-seq) to validate this finding. In addition, ChIP-seq and assay for transposase-accessible chromatin with high-throughput sequencing (ATAC-seq) data from multiple histone modifications were also derived from the Cistrome Data Browser and TCGA databases, respectively.

Chromloops is a novel, multispecies, protein-mediated chromatin loops database with many ChIA-PET, HiChIP and PLAC-Seq datasets that are available for studying the regulation of chromatin interactions [28]. The human HT-1376 ChIA-PET dataset was selected, and RAD21 was chosen as the ChIP-seq marker. Chromloops was used to visualise all the abovementioned ChIP-seq and ATAC-seq data.

### 2.7 Prediction of drug sensitivity and molecular docking for ESPL1

We predicted the relationship between ESPL1 expression and the half-maximal inhibitory concentration (IC50) of drugs in BC patients using the ‘oncoPredict’ package [29]. The drug sensitivity data were obtained from the Genomics of Drug Sensitivity in Cancer (GDSC) project (v2.0). We downloaded the crystal structure of ESPL1 (PDB ID: 7NJ1) from the RCSB PDB database and the 2D structures of the drug molecules from the PubChem database. ChemBio3D Ultra was used to perform free energy minimisation. The protein and drug molecular structures were processed and saved as.pdbqt files using AutoDockTools software. Finally, molecular docking was completed using QuickVina-w software in a Linux environment. The molecular docking results were presented in affinity, and PyMOL and Discovery Studio were used to visualise the docking models.

### 2.8 Prediction of upstream miRNA regulatory factors for ESPL1

We used four online tools to predict the upstream miRNAs that regulate ESPL1: miRmap, miRWalk, mirDIP and DIANA. The miRmap was used to show the miRNA and ESPL1 binding sites. We then used Cytospace software to construct a TF–target gene–miRNA regulatory network that included the predicted TF. Negative and positive likelihood ratio analyses were performed to evaluate the accuracy and reliability of miR-299-5p in distinguishing BC.

### 2.9 Statistical analysis

The standardised mean difference (SMD) and Wilcoxon Rank Sum Test were used to compare the groups. When the heterogeneity between datasets was excessive (*I*^2^ > 50 % and *p* ≤ 0.05), we calculated the SMD using a random-effects model. The 95 % confidence interval (CI) did not contain a 0 value, suggesting that the SMD results were statistically significant. A *t*-test was used to compare the two-sample means of the clinical characteristics, and an ANOVA was used to compare the multisample means. The Egger's test results, with *p* > 0.1, indicated that there was no publication bias in the SMD results. Sensitivity analysis was used to assess the stability of the results. Receiver operator characteristic (ROC) curves and summary receiver operator characteristic (sROC) curves were plotted separately, and the area under the curve (AUC) was used to determine the ability of the target to distinguish between BC samples and normal samples.

**Fig. 1:**
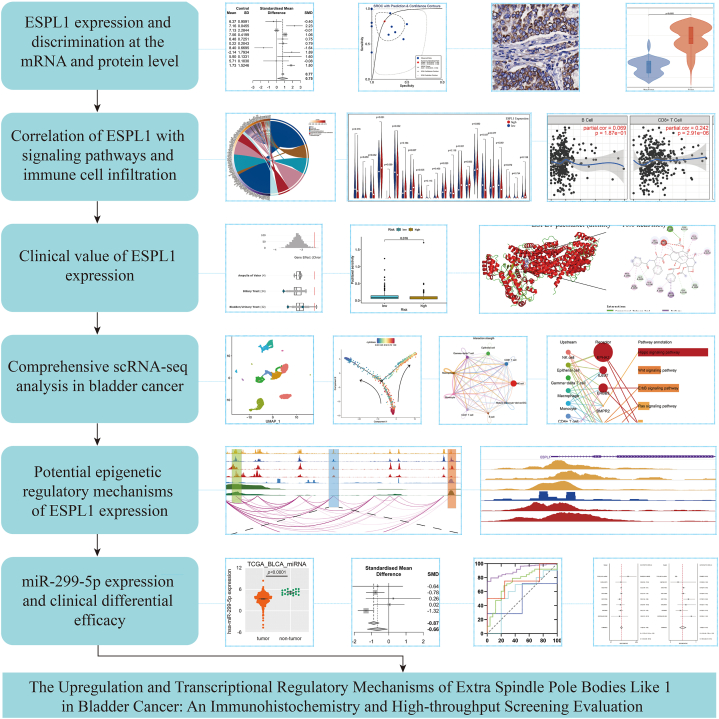
The research overflow of the present study.

In the present study, a *p*-value <0.05 indicated statistical significance. Stata (v18.0) was used to perform Egger's tests and sensitivity analyses and to produce scatter plots and sROC curves. GraphPad Prism 10.0 was used to plot the ROC curves, and the remainder of the analyses were performed using R (v4.3.1). The methodology used in the current study is illustrated in Fig. 1.

## 3 Results

### 3.1 Expression of ESPL1 in BC tissues

**Fig. 2:**
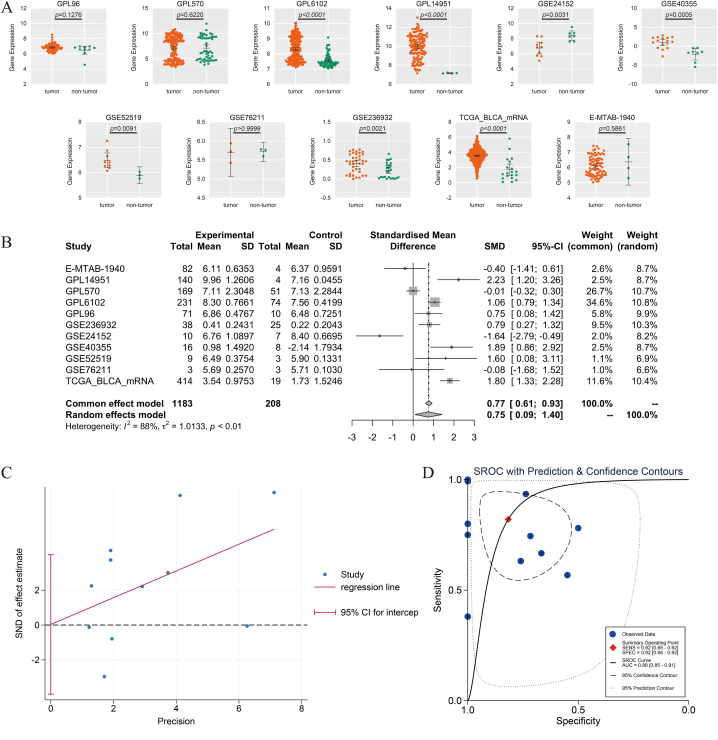
Comparison of ESPL1 messenger RNA (mRNA) expression levels in BC and control tissues. (A) Scatter plots of ESPL1 mRNA expression in each dataset. (B) Forest plot of ESPL1 mRNA expression. (C) Egger's test was used to detect publication bias. (D) Summary receiver operator characteristics.

Among the 11 datasets included, seven were found to have statistically significantly higher ESPL1 mRNA expression in the cancer group (*p* < 0.05; Fig. 2A). Considering the cohort's heterogeneity (*I*^2^ = 88 %, *p* < 0.01), a random-effects model was used to calculate the combined SMD for all the datasets. The random-effects model results showed that the ESPL1 mRNA expression in the cancer groups was higher overall than that in the control groups (SMD = 0.75; 95 % CI = 0.09, 1.40; Fig. 2B). The Egger's test results suggested that there was no publication bias in the included datasets (*p* = 0.984; Fig. 2C). ROC curves were plotted for a single cohort, and the AUC showed that ESPL1 expression could be used to distinguish between cancer and control tissues (Fig. S2). The combined sROC curves for all datasets corroborated this view (AUC = 0.88 [0.85–0.91]; Fig. 2D), suggesting that ESPL1 expression has significant potential in BC discrimination.

**Fig. 3:**
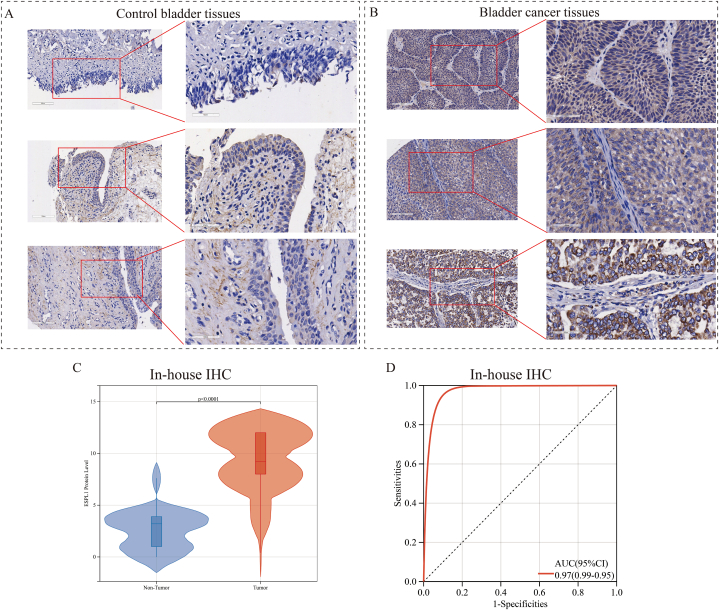
ESPL1 expression in BC and control tissues at the protein level.

To determine the expression of ESPL1 in BC at the protein level and verify the differential expression observed at the mRNA level, we subjected in-house samples to IHC staining. Under the microscope, we could clearly detect the presence of ESPL1 in internal BC tissues but not in normal tissues (Fig. 3A and B). Similarly, moderate-intensity IHC staining was detected in THPA BC tissues, whereas no staining was detected in normal tissues (Fig. S3). The results presented in Fig. 3C and **D** showed that significantly more ESPL1 was present in the tumour samples compared with the control samples (*p* < 0.0001), and the ROC curve showed that ESPL1 possessed an extremely strong discriminatory ability in BC tissues (AUC = 0.97 [0.99–0.95]). In addition, we combined the included datasets and in-house IHC data for further analyses. According to the results in Fig. S6, ESPL1 expression possessed high sensitivity (SENS = 0.82 [0.67–0.91]) and specificity (SPEC = 0.84 [0.68–0.93]). Combining the results of the negative likelihood ratio (DLR NEG = 0.21 [0.10–0.43]) and positive likelihood ratio (DLR POS = 5.09 [2.29–11.33]), ESPL1 expression was able to distinguish BC patients with higher precision.

### 3.2 Enrichment analysis of ESPL1 in BC

**Fig. 4:**
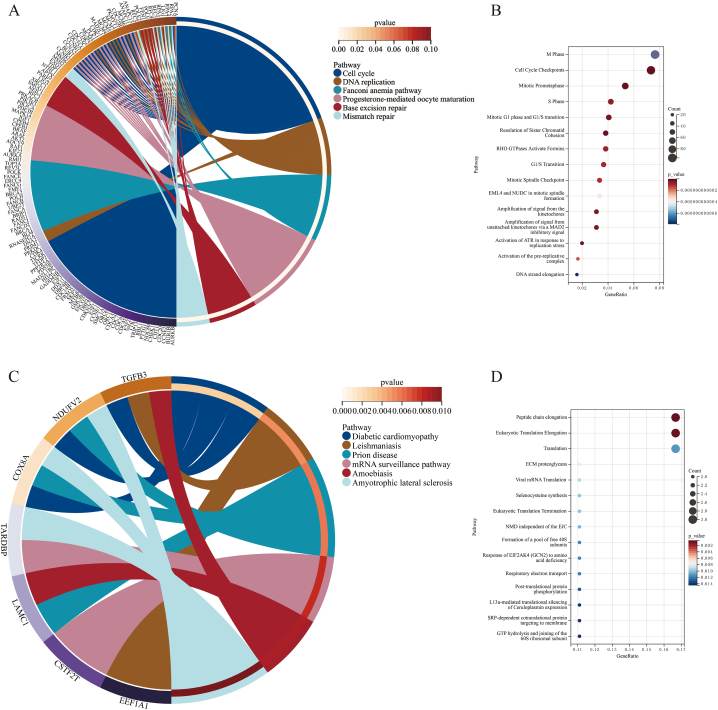
Pathway enrichment analysis of ESPL1 coexpressed genes (CEGs). (A) KEGG analysis of positively correlated ESPL1 CEGs. (B) Reactome analysis of positively correlated ESPL1 CEGs. (C) KEGG analysis of negatively correlated ESPL1 CEGs. (D) Reactome analysis of negatively correlated ESPL1 CEGs.

An analysis of genes coexpressed with ESPL1 yielded 2020 positively (*r* ≥ 0.6, *p* < 0.05) and 31 negatively (*r* ≤ 0.6, *p* < 0.05) correlated CEGs. Enrichment analysis showed that the positively correlated CEGs were mainly involved in cell cycle- and DNA replication-related signalling pathways (Fig. 4A), with similar results obtained when the GO (Fig. S4A) and Reactome (Fig. 4B) databases were used. A KEGG analysis showed that the negatively correlated CEGs were mainly enriched in signalling pathways (e.g., diabetic cardiomyopathy and mRNA surveillance pathway) (Fig. 4C), whereas the GO terms (Fig. S4B) and Reactome analysis (Fig. 4D) suggested that the negatively correlated CEGs were mainly involved in regulating intracellular protein synthesis and function.

### 3.3 Significance of ESPL1 expression in the tumour microenvironment (TME)

**Fig. 5:**
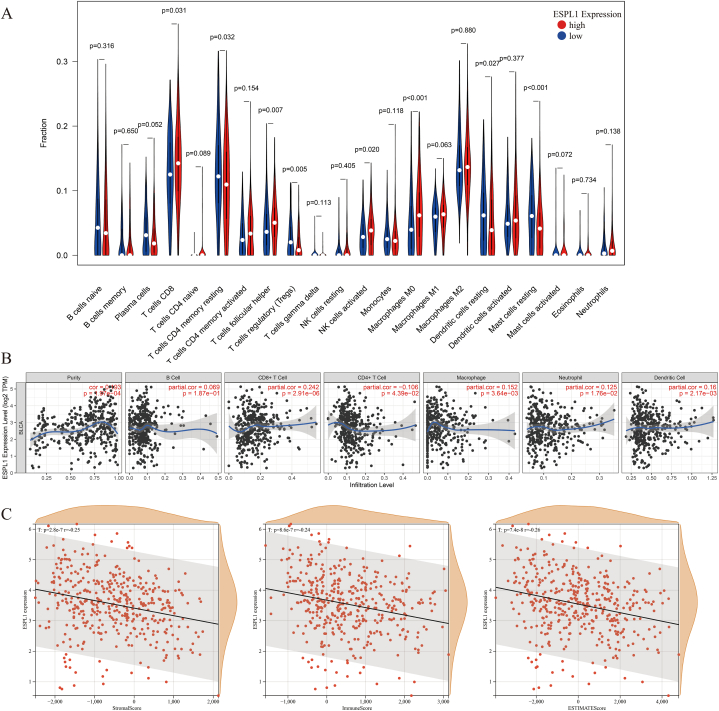
There was a close association between ESPL1 expression and BC tumour microenvironment. (A) Differences in immune cell levels between the high and low ESPL1 expression groups. (B) Correlation between ESPL1 expression and immune cells. (C) Immune infiltration analysis of ESPL1 expression.

The immune response plays an essential role in preventing tumorigenesis, tumour cell proliferation and tumour metastasis. Hence, we utilised different algorithms to investigate the association between ESPL1 expression and the tumour microenvironment (TME) from multiple perspectives. Using the CIBERSORT algorithm, we found significant differences between the high- and low-ESPL1-expression groups in terms of the levels of eight types of immune cells (*p* < 0.05; Fig. 5A). For example, the results indicated that CD8^+^ T-cell levels were higher in the high-expression group's samples than in the low-expression group's samples (*p* = 0.031). The results obtained using the TIMER algorithm showed that ESPL1 upregulation resulted in tumour purity and elevated levels of four types of immune cells (CD8^+^ T cells, neutrophils, macrophages and dendritic cells) (*ρ* > 0, *p* < 0.05; Fig. 5B), while the opposite was indicated for CD4^+^ T cells (*ρ* < 0, *p* < 0.05; Fig. 5B). In addition, the results of the ESTIMATE algorithm suggested that high ESPL1 expression was significantly negatively correlated with the overall immune infiltration score of the TME (*r* < 0, *p* < 0.05; Fig. 5C).

### 3.4 Analysis of ESPL1 expression and cellular communication in multiple cells

**Fig. 6:**
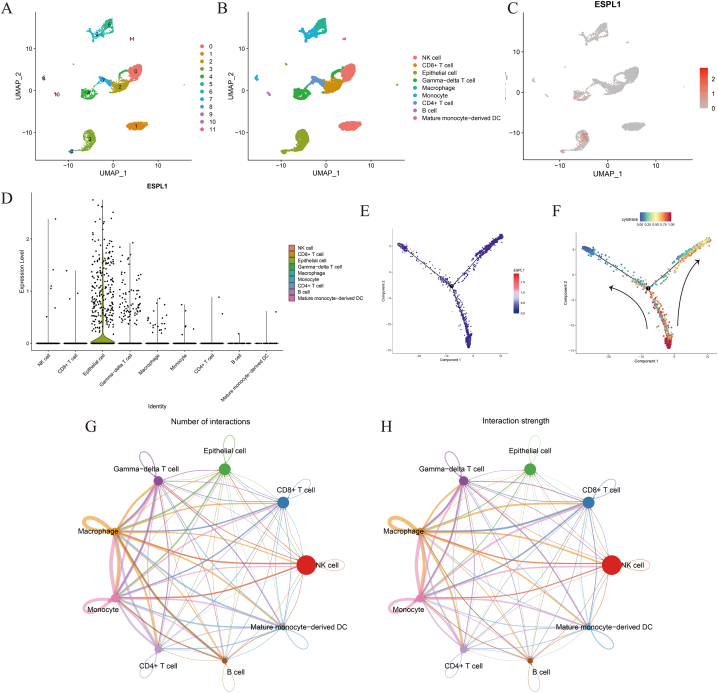
Comprehensive analysis of single-cell sequencing (A) Included cells were categorised into 12 taxa. (B) All cells were annotated into nine cell types. (C) UMAP plot showing elevated ESPL1 expression in epithelial cells. (D) Violin plot of ESPL1 expression in different cell types. (E) Expression levels of ESPL1 during cell differentiation. (F) Time-dependent analysis of cell differentiation trajectories. (G) Number of cell cluster interactions based on the “Cellchat” package. (H) Strength of cell cluster interactions based on the “Cellchat” package.

Considering the positive significance of ESPL1 in the TME, we further explored ESPL1 expression in single cells, along with the interconnections between the cells. We screened 5201 cells that were annotated and classified into nine cell types (Fig. 6A and B). The UMAP plots showed that ESPL1 was predominantly expressed in BC epithelial cells (Fig. 6C and D) and that ESPL1 expression tended to rise with cell differentiation (Fig. 6E and F). The results of our Cellchat analysis suggested that epithelial cells exist in a close cellular communication network with other cells (Fig. 6G and H).

**Fig. 7:**
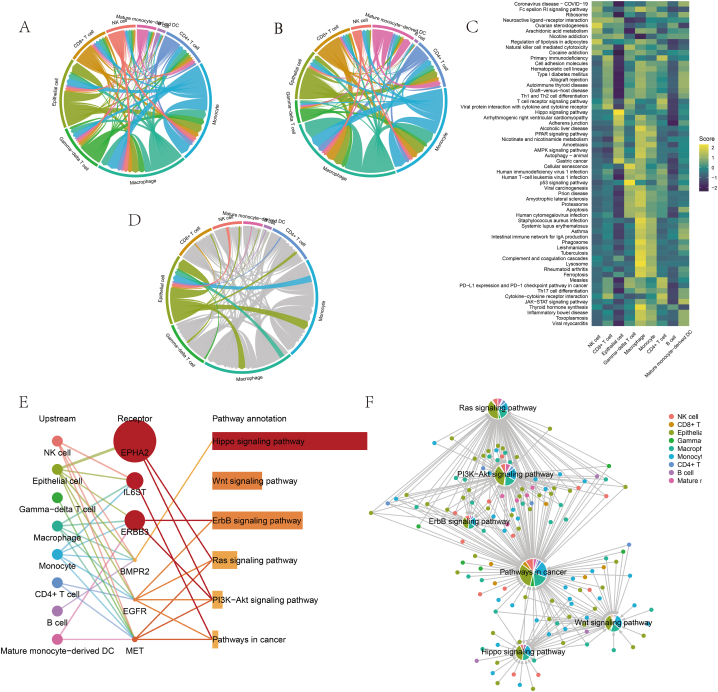
Cell communication analysis based on the “Commpath” package. (A) Number of interactions between different cell clusters. (B) Interaction strength between different cell clusters. (C) Thermogram of the differentially activated pathways in each cell cluster. (D) Signalling pathways and associated functional ligand–receptor pair interactions between epithelial cells and the remaining cell clusters. (E) Major activation pathways and associated receptors of epithelial cells with the remaining cell clusters. (F) A network diagram was used to visualise significantly upregulated signalling pathways in epithelial cells.

Functional ligand–receptor pairs were screened using the Commpath package, and the signalling analysis was completed. The results showed that the number of interactions and total interaction strength between different cell clusters were extremely high (Fig. 7A and B), and there were differences in the degree of activity in specific signalling pathways (Fig. 7C). We focused on epithelial cells and demonstrated their signalling pathways and associated functional ligand–receptor pair interactions (Fig. 7D). As shown in Fig. 7E and **F**, we found that epithelial cells were involved in the Hippo, ErbB, PI3K-Akt and Ras signalling pathways mainly through their binding to the following receptors: EPHA2, IL6ST, ERBB3, BMPR2, EGFR and MET.

### 3.5 Regulatory mechanisms of elevated ESPL1 expression

**Fig. 8:**
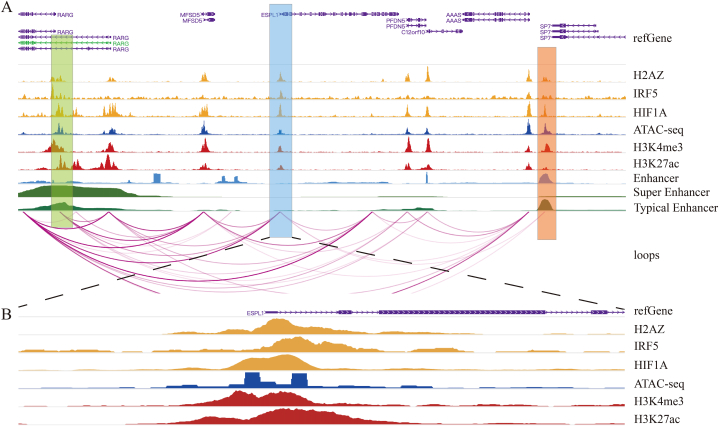
Exploring potential mechanisms of ESPL1 expression through epigenetic regulation. (A) Transcriptional regulation of ESPL1 in BC. (B) Magnified blue shaded region.

Next, we undertook an in-depth study of the epigenetic regulatory mechanism of ESPL1. The three TFs with the highest prediction scores were H2AZ, IRF5 and HIF1A, and we examined the likelihood that these TFs bound at the upstream TSS of ESPL1 to regulate ESPL1 expression. ChIP-seq of these TFs was performed, and the results verified this notion (blue shaded area in Fig. 8). The ATAC-seq peak was clearly seen upstream in the ESPL1 sequence, suggesting open chromatin regions near the ESPL1 TSS. Histone modifications could be used to label active promoters (H3K27ac, H3K4me3), and the presence of H3K27ac and H3K4me3 peaks at the ESPL1 TSS suggested the presence of a nearby promoter. In addition, loops were found between the promoter of the ESPL1 TSS and upstream super-enhancers (SEs) and downstream typical-enhancers (TEs), indicating that the promoter might have direct contact and interact with the enhancers, boosting the transcriptional activity of ESPL1 (located in the green- and red-shaded areas in Fig. 8, respectively). Given that RAD21 was a ChIP marker, it might mediate the promoter–SE loops and promoter–TE loops and enhance the expression of ESPL1.

### 3.6 Correlation between ESPL1 expression and drug sensitivity in BC patients

**Fig. 9:**
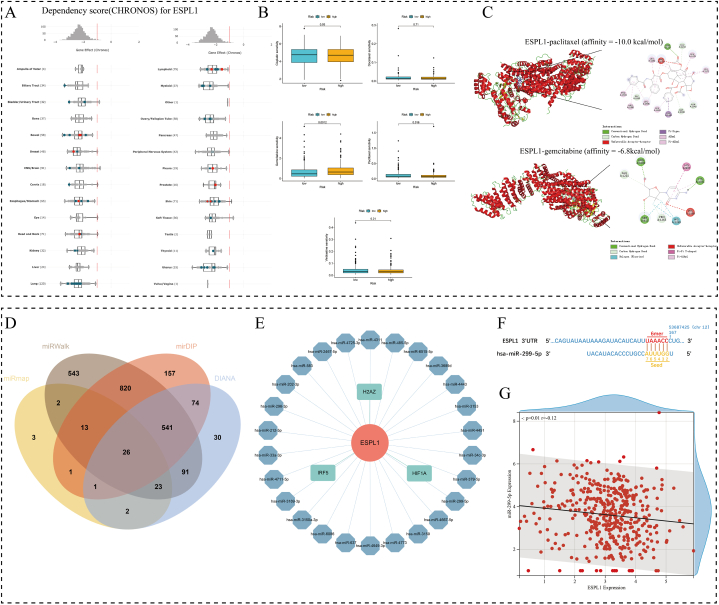
ESPL1 expression clinical significance and prediction of upstream microRNA (miRNA) regulators. (A) Dependence of multiple tumours on ESPL1 expression. (B) Relationship between high and low ESPL1 expression and drug sensitivity. (C) Molecular docking with paclitaxel and gemcitabine based on the ESPL1 protein. (D) Venn plots of the prediction results of the four miRNA prediction tools. (E) TFs–target gene–miRNA regulatory network. (F) Binding sites of ESPL1 and miR-299-5p. (G) Correlation between ESPL1 and miR-299-5p expression.

We also analysed the importance of ESPL1 in BC and whether it was associated with drug sensitivity in BC patients. We first examined the dependence of 1095 tumour cell lines on ESPL1; the results suggested that ESPL1 was indispensable for the growth and reproduction of all the examined cancer cell lines, including BC cell lines (chronons score < −1; Fig. 9A). The cancer-promoting effects of ESPL1 were also confirmed by analyses of 27 common BC cell lines (Fig. S5). Then, we analysed the relationship between ESPL1 mRNA expression and various clinical characteristics of BC patients, finding that ESPL1 expression was significantly correlated with patient age and tumour grade (*p* < 0.05; Table 2). Finally, we conducted a drug sensitivity analysis and found that high ESPL1 expression was associated with an elevated IC50 of paclitaxel (p = 0.016) and a low IC50 of gemcitabine (p = 0.0012) in BC patients (Fig. 9B). Molecular docking experiments showed that ESPL1 had a high molecular affinity for paclitaxel (−10.0 kcal/mol) and gemcitabine (−6.8 kcal/mol), suggesting that these drugs play vital roles in treating BC (Fig. 9C).

**Table 2:** Investigating the relationship between ESPL1 mRNA expression and clinical characteristics in patients with ESPL1 from the TCGA_BLCA_mRNA dataset.

Clinical features	ESPL1 mRNA Expression			*t* (*t*-test) or *F*(ANOVA test)	*p* value
	Number	Mean	SD		
Age				2.411	0.0163
>60	315	3.182	0.9637		
≤60	112	2.920	1.055		
Sex				1.276	0.2027
Male	311	3.151	1.004		
Female	116	3.013	0.9650		
BMI				0.3409	0.7333
≥30	86	3.085	1.036		
＜30	279	3.127	0.9747		
Race				1.730	0.1786
White	341	3.103	1.039		
Asian	44	3.051	0.8993		
Black or African American	24	3.292	0.7342		
Clinical stage				1.335	0.2626
Stage I	2	1.783	1.493		
Stage II	134	3.086	0.9847		
Stage III	147	3.158	0.9873		
Stage IV	142	3.100	1.002		
Grade				0.3563	0.0004
High Grade	403	3.148	0.9761		
Low Grade	21	2.367	1.050		
Diagnosis_subtype				1.859	0.0638
Non-Papillary	285	3.168	0.9968		
Papillary	137	2.977	0.9824		
Lymphovascular_invasion_present				1.590	0.1128
No	133	3.239	0.9657		
Yes	160	3.055	1.002		
Smoker				0.03775	0.9699
No	194	3.115	0.9566		
Yes	233	3.112	1.026		
Primary_therapy_outcome_success				0.2213	0.8815
CR	154	3.188	0.9389		
PR	16	3.229	0.9063		
PD	43	3.085	0.9108		
SD	22	3.272	1.285		
Radiation_therapy				0.1486	0.8820
No	268	3.173	0.9829		
Yes	10	3.220	0.3110		
Pathologic_M				0.3545	0.7233
M0	206	3.118	1.014		
M1	11	3.006	1.068		
Pathologic_N				0.3695	0.7750
N0	248	3.136	0.9860		
N1	49	3.111	1.010		
N2	76	3.094	0.9716		
N3	8	2.771	1.270		
Pathologic_T				1.144	0.3196
T1&T2	127	3.052	0.9923		
T3	205	3.152	0.9832		
T4	61	2.944	1.066		

### 3.7 miR-299-5p was predicted to be an upstream regulator of ESPL1

Considering the importance of miRNAs in tumour development and early diagnosis of cancer, we utilised four online tools to predict the upstream miRNAs regulating ESPL1. We intersected the prediction results to obtain 26 target miRNAs (Fig. 9D) and combined them with the three predicted TFs described in the previous section to construct a TF–target gene–miRNA regulatory network (Fig. 9E). Furthermore, we selected miR-299-5p for subsequent analysis. miRmap was used to identify the binding sites of ESPL1 and miR-299-5p (Fig. 9F), and the correlation analysis suggested that ESPL1 and miR-299-5p were significantly negatively correlated (*r* = −0.12, *p* = 0.01; Fig. 9G).

### 3.8 miR-299-5p showed low expression in BC blood and tissue samples

**Fig. 10:**
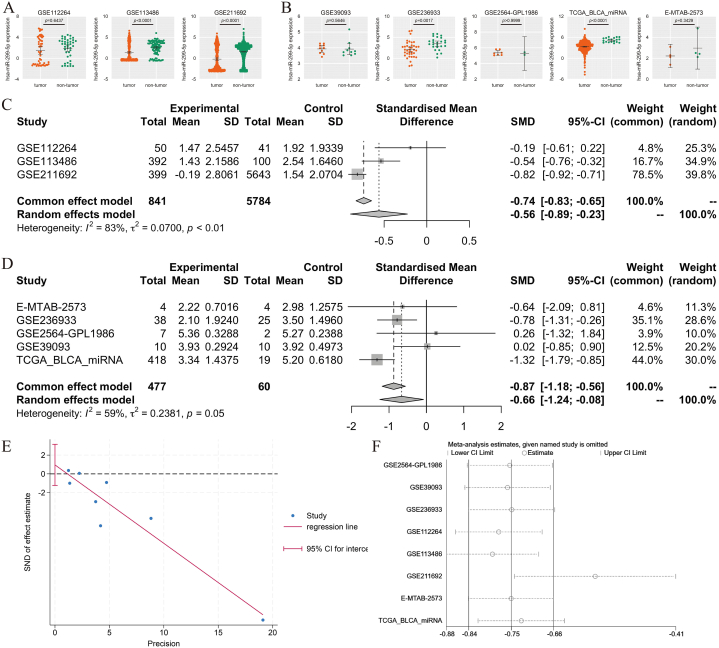
Expression levels of miR-299-5p in blood and tissue samples in the BC and control groups. (A) Scatter plots based on blood samples. (B) Scatter plots based on tissue samples. (C) Forest plot based on blood samples. (D) Forest plot based on tissue samples. (E) Sensitivity analysis. (F) Egger's test.

An analysis of blood and tissue miRNA datasets from BC patients showed that miR-299-5p expression was downregulated (*p* < 0.05; Fig. 10A and B). The results were not statistically significant in some of the datasets, and this was somewhat related to the small number of samples within the corresponding datasets. Moreover, we calculated the integrated SMD in the blood and tissue samples separately using a random-effects model, and the results showed that the expression of miR-299-5p was downregulated in both types of samples (blood SMD = −0.56 and 95 % CI = −0.89, −0.23; tissue SMD = −0.66 and 95 % CI = −1.24, −0.08; Fig. 10C and D). Egger's test results indicated that there was no publication bias (*p* = 0.327; Fig. 10E). The sensitivity analysis results showed considerable heterogeneity among the datasets (Fig. 10F). Considering that the data came from different platforms and were heterogeneous, it was appropriate to utilise a random-effects model to calculate the SMD.

### 3.9 miR-299-5p possessed high detection efficacy in both blood and tissue samples from BC patients

**Fig. 11:**
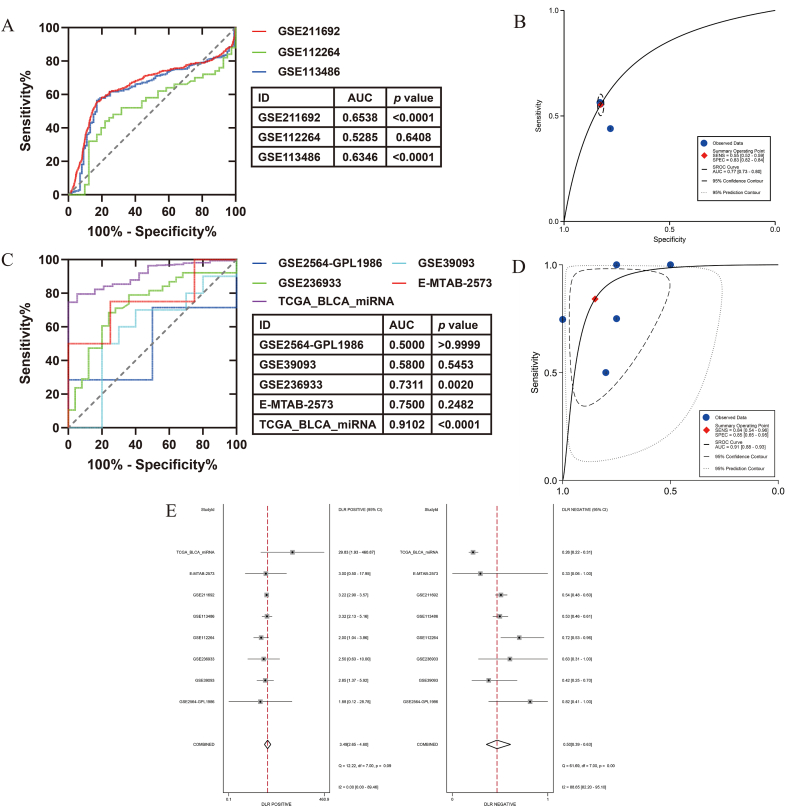
Detection efficacy of miR-299-5p expression in BC blood and tissue samples. (A) Receiver operator characteristic curves (ROC) curves based on blood samples. (B) Summary receiver operator characteristic curve (sROC) based on blood samples. (C) ROC curves based on tissue samples. (D) sROC curve based on tissue samples. (E) Positive likelihood ratio and negative likelihood ratio analysis.

The differential expression of miR-299-5p was observed in blood and tissue samples from BC patients, implying its immeasurable potential for the clinical detection of BC. The ROC and sROC showed that BC could be detected by assessing miR-299-5p expression in blood samples (AUC = 0.77 [0.73–0.80]; Fig. 11A and B) and tissue samples (AUC = 0.91 [0.88–0.93]; Fig. 11C and D). Furthermore, we analysed the negative and positive likelihood ratios for the combined sample types. The results showed a positive likelihood ratio of 3.49 with a 95 % CI of 2.65–4.60 and a negative likelihood ratio of 0.5 with a 95 % CI of 0.39–0.63 (Fig. 11E). These results suggested that miR-299-5p expression could be used to accurately exclude and distinguish BC and had a positive impact on the sensitivity of the distinction.

## 4 Discussion

This was the first comprehensive study on ESPL1 expression in BC. It involved the collection and analysis of 1183 BC and 208 noncancer samples. First, there was a significant upregulation of ESPL1 mRNA in the BC samples. We verified this finding at the protein level via IHC experiments. Second, we determined the expression of ESPL1 in different cell clusters and its relationship with cell differentiation using scRNA-seq analysis. Third, we utilised an epigenomic approach to identify the potential regulatory mechanisms of ESPL1 expression. Fourth, we simultaneously investigated the associations between ESPL1 expression and immune cell infiltration, potential signalling pathways, cell communication and drug sensitivity in BC patients. Finally, we predicted that ESPL1 was a downstream target gene of miR-299-5p and that the low expression of miR-299-5p in blood and tissue samples had excellent discriminatory efficacy for BC.

Previous studies have shown that ESPL1 is highly expressed in various tumours. Song et al. [11] showed that ESPL1 expression was upregulated in HCC and substantially increased the mortality rate of HCC patients. Liu et al. [30] suggested that the high expression of ESPL1 promoted the proliferation, migration and apoptosis of LUAD cells. Gui et al. [31] also found that ESPL1 expression was significantly elevated in ovarian cancer and correlated with poor prognosis. Although Chen et al. previously reported the presence of upregulated ESPL1 expression in BC, they did not explore ESPL1 expression in sufficient depth and were limited in their analyses by the small number of included samples (n = 246) and a lack of in-house samples for experimental validation [15]. In contrast, we identified elevated ESPL1 expression in BC using a large number of samples (n = 1391) and validated this finding at the protein level via IHC staining of in-house samples (n = 202). Furthermore, we used scRNA-seq to determine that ESPL1 was predominantly distributed in BC epithelial cells and was continuously expressed at elevated levels with cell differentiation.

Increasing evidence indicates that ESPL1 participates in cell cycle signalling in breast cancer [32], endometrial cancer (UCEC) [33] and HCC [11], thus promoting the proliferation and migration of cancer cells [14]. However, in terms of BC, the potential pathways in which ESPL1 is involved still need to be better defined. ESPL1 expression was the highest in the S and G2 phases of cancer cells, suggesting that ESPL1 might promote mitosis and tumour proliferation by regulating these phases of the cancer cell cycle [11]. The literature has shown that activated ESPL1 released endoglin subunits (e.g., RAD21) and promoted the provision of sister chromatid cohesion (SCC) to ensure stable inheritance [33]. A knockdown of ESPL1 might lead to G1-S transition arrest and SCC interruption, ultimately leading to cell death [34]. In BC, ESPL1 was similarly thought to influence BC cell proliferation and differentiation by participating in SCC and segregation [35]. Based on our further exploration of cellular communication, we concluded that epithelial cells mainly bound to receptors such as EPHA2, IL6ST, ERBB3, BMPR2, EGFR and MET and participated in cancer cell proliferation, apoptosis and differentiation through the Hippo, ErbB, PI3K-Akt and Ras signalling pathways. The results of the present study indicated that both positively and negatively correlated CEGs of ESPL1 were involved in BC cell cycle–related signalling pathways. Therefore, we hypothesised that ESPL1 and CEGs influence the cell cycle by participating in cell division and promote the proliferation and differentiation of BC epithelial cells through complex cell-to-cell communication.

When we explored the association between ESPL1 expression and the TME, interesting findings emerged. The TME composed of immune and nonimmune cells (e.g., endothelial cells), with the former accounting for the vast majority of the TME cell population [36]. The literature suggests that the composition of the TME was closely associated with tumour progression, immune evasion and the success of immunotherapy [13]. The present found that the TME composition was significantly different between the high- and low-ESPL1-expression groups and that ESPL1 expression correlated with the levels of various immune cells. Meanwhile, the overexpression of ESPL1 was significantly associated with higher tumour purity and lower immune cell levels in TME. In previous studies, ESPL1 expression in LUAD was positively correlated with the level of infiltration of Th2 cells and negatively correlated with the level of infiltration of mast cells, iDCs, DCs and CD8T cells and might be involved in immune evasion and tumour migration [13]. However, there is still a gap in the mining of ESPL1 in the field of immunity, especially in BC. After combining the findings emerging from multiple immunoassay algorithms, we concluded that ESPL1 might serve as a novel immune cell infiltration-associated marker for BC.

Given the poor prognosis associated with BC, we considered the clinical value of ESPL1 from several perspectives. First, a previous study reported that ESPL1 was an effective predictor of overall survival, recurrence-free survival and metastasis-free survival in prostate cancer patients [37], and similar findings have been reported for UCEC [33,38]. Here, we determined the importance of ESPL1 in BC cells based on CRISPR-Cas9 functional deletion screening data provided by the Depmap Portal. In addition, we found that ESPL1 possessed excellent discriminatory efficacy and was strongly correlated with BC patients’ age and tumour grade. Second, although cisplatin-based chemotherapy has been shown to be effective against perioperative and first-line metastatic disease, the treatment options for BC are still evolving [39]. For example, immune checkpoint inhibitors have been used to treat platinum-refractory BC, predominantly atezolizumab and pembrolizumab [40,41]. Metformin also contributed to the inhibition of BC activity and its induction of BC apoptosis [42]. It was previously reported that paclitaxel could be combined with obatoclax to induce apoptosis and alleviate paclitaxel resistance in uroepithelial cancer cells [43]. In combination with radiotherapy and chemotherapy, gemcitabine has been shown to be useful in the systemic treatment of BC, whether muscle-invasive BC or nonmuscle-invasive BC [[44], [45], [46], [47]]. However, few studies have reported the effect of ESPL1 expression on chemotherapy in BC patients. In the present study, we found that high ESPL1 expression was closely associated with the IC50 of paclitaxel and gemcitabine in BC patients. As a BC oncogene, ESPL1 has potential as a novel BC diagnostic biomarker. Meanwhile, elevated ESPL1 expression could affect the sensitivity of BC to paclitaxel and gemcitabine, improving therapeutic efficacy.

Although the significance of high ESPL1 expression in BC has been clarified, the underlying regulatory mechanism responsible for the elevated expression remains unknown. Three-dimensional genomics has been increasingly used to reveal genomic functions and regulatory mechanisms through studying the spatial structures and interactions of chromosomes [48]. Chromatin loops are essential structures in the three-dimensional conformation of chromatin because they may be able to bring distant gene regulatory elements and regions close together, thus influencing gene expression [49,50]. In the current study, we first identified chromatin open regions in the TSS of ESPL1 using ATAC-seq and verified that H2AZ, IRF5 and HIF1A could act as upstream TFs to regulate the expression of ESPL1 using ChIP-seq. In addition, utilising histone modification ChIP-seq and high-throughput chromosome conformation capture (Hi-C) data, we found that, because of the presence of loops, the ESPL1 TSS was near upstream SEs and downstream TEs, which were mediated by RAD21 and, thus, potentially enhanced ESPL1 expression. This might be related to the release of the RAD21 subunit of the chromatin-binding protein by ESPL1, which, in turn, enables the segregation of sister chromatids [33,51]. Although previous studies had repeatedly explored the elevated expression of ESPL1 in various tumours, this was the first time that the epigenetic regulatory mechanisms of ESPL1 have been analysed using histone modification and three-dimensional genomics with the results being reported. Hence, our findings filled a considerable gap in the field, which was a strength of the current study.

miRNAs are often considered biomarkers for tumour suppression, oncogenic factors and diagnosis of cancer progression [52]. In recent years, the early diagnosis of tumours based on miRNA levels in body fluid samples (blood, saliva and urine) has received much attention [53,54]. miR-299-5p is located in the imprinted Dlk1-Dio3 region on chromosome 14q32.31 [55]. Previous studies have shown that miR-299-5p is expressed at a low level in CRC, thyroid and breast cancers [56,57] and at a high level in osteosarcoma [58] and that it could be used as an early diagnostic marker for a wide range of cancers [56,59]. Here, we examined 6625 blood samples and 537 tissue samples from BC patients, finding, for the first time, that miR-299-5p expression was downregulated in the blood and tissues of BC patients. miR-299-5p showed a strong ability to distinguish between normal individuals and BC patients in both sample types, suggesting that miR-299-5p was a potential marker for the early diagnosis of BC. In addition, introducing a blood-based noninvasive assay would greatly improve the early diagnosis rate of BC and promote the development of the cancer prevention field.

The results of the present study are promising; however, certain limitations still exist. Specifically, in vivo and in vitro assays must be developed to examine the biological function of ESPL1 and potential of BC as a predictive biomarker. In addition, more in-house high-throughput technologies are required for further studies to validate the potential epigenetic regulatory mechanisms of ESPL1.

## 5 Conclusion

In conclusion, in the present study, we utilised in-house samples and multicentre data to determine that ESPL1 expression was upregulated in BC and mainly distributed in BC epithelial cells. The elevated expression of ESPL1 was associated with TFs at the upstream TSS and distant regulatory elements of chromatin loops. ESPL1 might be an immune-related predictive and diagnostic marker of BC. The high expression of ESPL1 was found to play a cancer-promoting role and affect the drug sensitivity of BC patients. Finally, miR-299-5p was downregulated in the blood and tissues of BC patients, and it also has potential as a new early diagnostic marker for BC.

## Ethics approval and consent to participate

Studies concerning human subjects have been approved and supported by the Medical Ethics Review Committee of the First Affiliated Hospital of Guangxi Medical University(2022-KT-GUOJI-146). All patients who provided internal samples signed an informed consent form.

## Consent for publication

All authors have reviewed the manuscript and agreed to publication.

## Data availability statement

All data relevant to this study are available in public databases or in Supplementary Material 1, and do not need to be deposited in public databases. Any other data from this study can be obtained from the authors upon reasonable request.

## Funding

This study was supported by Guangxi Zhuang Autonomous Region Health Commission Self-financed Scientific Research Project (Z-A20230502), Innovation Project of Guangxi Graduate Education (JGY2023068), Guangxi Higher Education Undergraduate Teaching Reform Project (2022JGA146), Guangxi Educational Science Planning Key Project (2022ZJY2791), 10.13039/501100011827Guangxi Medical University Undergraduate Education and Teaching Reform Project (2023Z10).

## CRediT authorship contribution statement

**Wei Zhang:** Writing – original draft, Validation, Software, Conceptualization. **Zi-Qian Liang:** Writing – original draft, Software. **Rong-Quan He:** Visualization, Conceptualization. **Zhi-Guang Huang:** Data curation. **Xiao-Min Wang:** Visualization. **Mao-Yan Wei:** Visualization. **Hui-Ling Su:** Visualization. **Zhi-Su Liu:** Visualization. **Yi-Sheng Zheng:** Visualization. **Wan-Ying Huang:** Software, Data curation. **Han-Jie Zhang:** Resources, Funding acquisition. **Yi-Wu Dang:** Supervision, Conceptualization. **Sheng-Hua Li:** Resources, Methodology. **Ji-Wen Cheng:** Validation. **Gang Chen:** Writing – review & editing, Validation. **Juan He:** Writing – review & editing, Visualization, Supervision, Project administration.

## Declaration of competing interest

The authors declare that they have no known competing financial interests or personal relationships that could have appeared to influence the work reported in this paper.

## Figures and Tables

**Figure:**
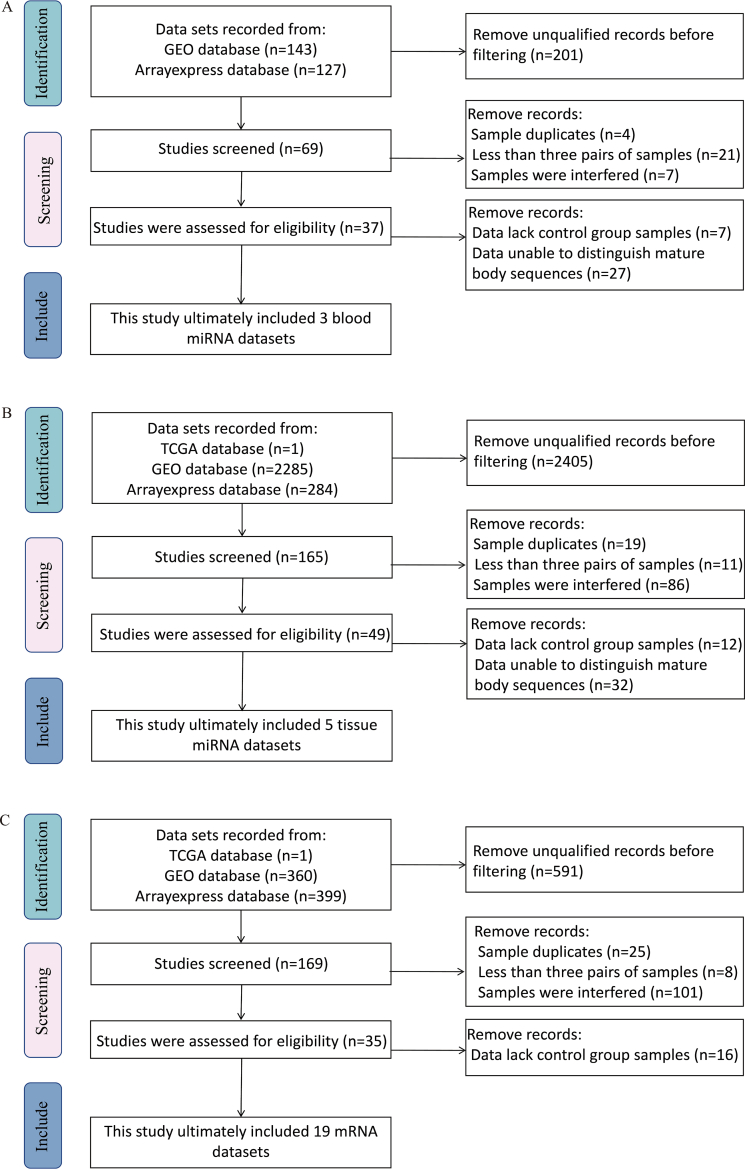
figs1

**Figure:**
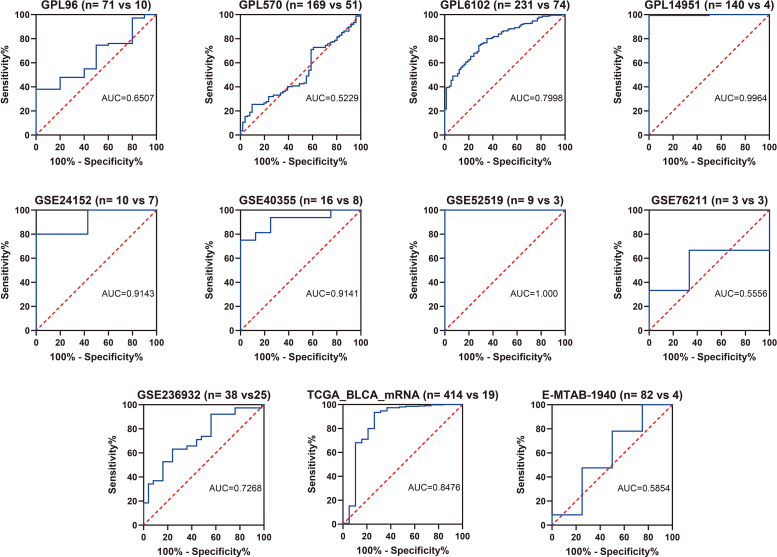
figs2

**Figure:**
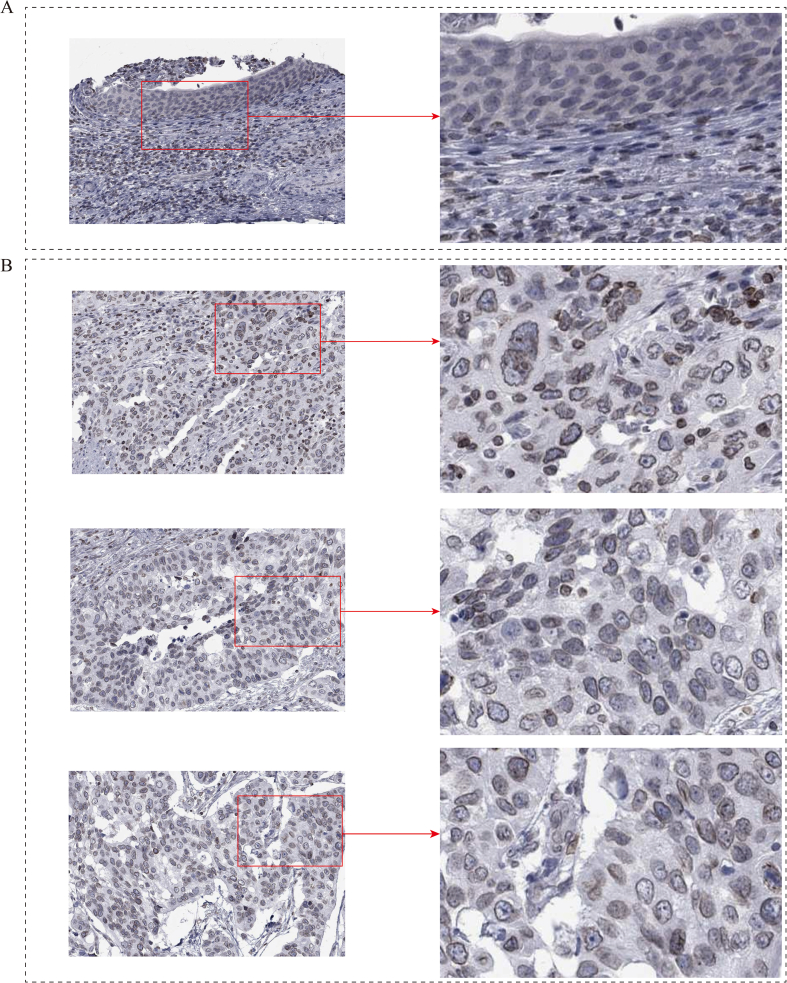
figs3

**Figure:**
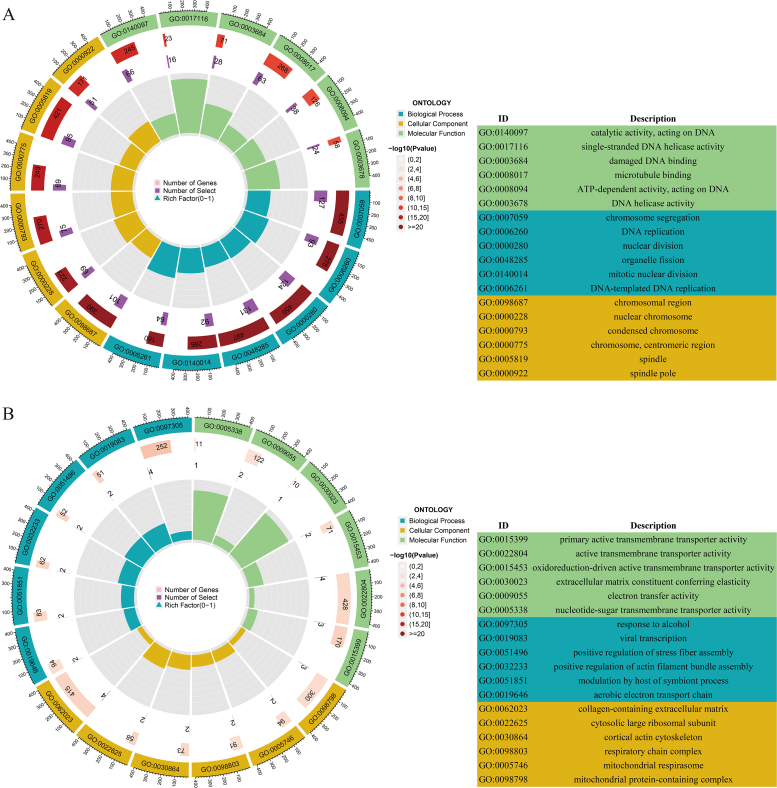
figs4

**Figure:**
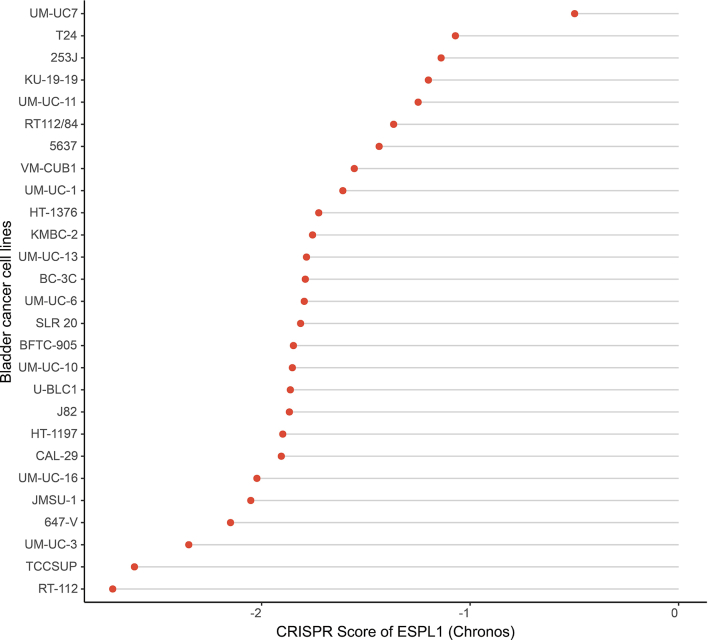
figs5

**Figure:**
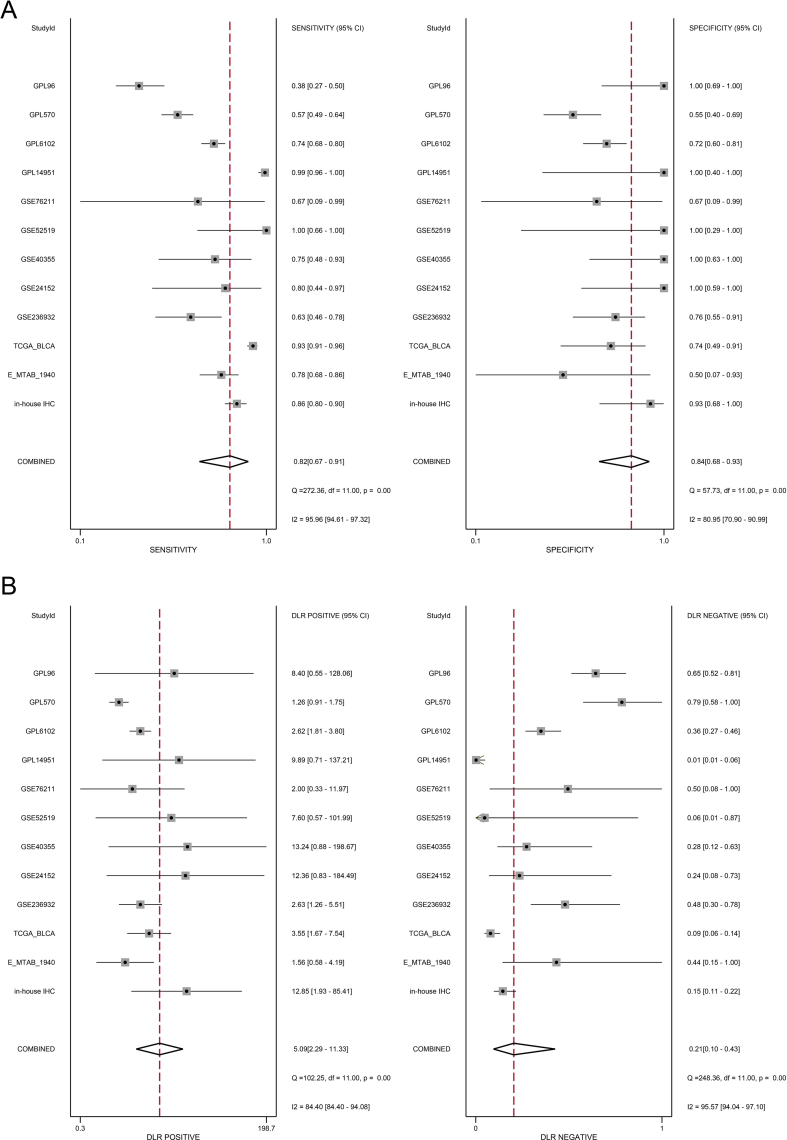
figs6

## References

[1] Lobo N. (2022). Epidemiology, screening, and prevention of bladder cancer. Eur Urol Oncol.

[2] Sung H. (2021). Global cancer Statistics 2020: GLOBOCAN Estimates of incidence and mortality worldwide for 36 cancers in 185 countries. CA Cancer J Clin.

[3] Lai H. (2021). Single-cell RNA sequencing reveals the epithelial cell heterogeneity and invasive subpopulation in human bladder cancer. Int. J. Cancer.

[4] Huang L., Xie Q., Deng J., Wei W.F. (2023). The role of cancer-associated fibroblasts in bladder cancer progression. Heliyon.

[5] Sun Z. (2023). FTO promotes proliferation and migration of bladder cancer via enhancing stability of STAT3 mRNA in an m6A-dependent manner. Epigenetics.

[6] Farouk S.M., Khafaga A.F., Abdellatif A.M. (2023). Bladder cancer: therapeutic challenges and role of 3D cell culture systems in the screening of novel cancer therapeutics. Cancer Cell Int..

[7] Dyrskjot L. (2023). Bladder cancer. Nat Rev Dis Primers.

[8] Su X., Dong C., Liao W., Liu W. (2023). Oncological effectiveness of bladder-preserving trimodal therapy versus radical cystectomy for the treatment of muscle-invasive bladder cancer: a system review and meta-analysis. World J. Surg. Oncol..

[9] Harsanyi S. (2022). Biomarkers of bladder cancer: cell-free DNA, epigenetic modifications and non-coding RNAs. Int. J. Mol. Sci..

[10] Konecna M., Abbasi Sani S., Anger M. (2023). Separase and roads to disengage sister chromatids during anaphase. Int. J. Mol. Sci..

[11] Song R. (2022). ESPL1 is elevated in hepatocellular carcinoma and predicts prognosis. Int. J. Gen. Med..

[12] Liu Z. (2021). ESPL1 is a novel prognostic biomarker associated with the malignant features of glioma. Front. Genet..

[13] Nie Z. (2022). Extra spindle Pole bodies-like 1 serves as a prognostic biomarker and promotes lung adenocarcinoma metastasis. Front. Oncol..

[14] Zhong Y. (2023). Pan-Cancer analysis and experimental validation identify the oncogenic nature of ESPL1: potential therapeutic target in colorectal cancer. Front. Immunol..

[15] Chen Q. (2019). Bioinformatics analysis identified key molecular changes in bladder cancer development and recurrence. BioMed Res. Int..

[16] Ramazi S., Daddzadi M., Sahafnejad Z., Allahverdi A. (2020). Epigenetic regulation in lung cancer. MedComm.

[17] Ritchie M.E. (2015). Limma powers differential expression analyses for RNA-sequencing and microarray studies. Nucleic Acids Res..

[18] Leek J.T., Storey J.D. (2007). Capturing heterogeneity in gene expression studies by surrogate variable analysis. PLoS Genet..

[19] Onishi I. (2021). To discover the efficient and novel drug targets in human cancers using CRISPR/cas screening and databases. Int. J. Mol. Sci..

[20] Wu T. (2021). clusterProfiler 4.0: a universal enrichment tool for interpreting omics data. Innovation.

[21] Yu G., He Q.Y. (2016). ReactomePA: an R/Bioconductor package for reactome pathway analysis and visualization. Mol. Biosyst..

[22] Zeng D. (2021). IOBR: multi-omics immuno-oncology biological research to decode tumor microenvironment and signatures. Front. Immunol..

[23] Hao Y. (2021). Integrated analysis of multimodal single-cell data. Cell.

[24] Aran D. (2019). Reference-based analysis of lung single-cell sequencing reveals a transitional profibrotic macrophage. Nat. Immunol..

[25] Gulati G.S. (2020). Single-cell transcriptional diversity is a hallmark of developmental potential. Science.

[26] Jin S. (2021). Inference and analysis of cell-cell communication using CellChat. Nat. Commun..

[27] Lu H. (2022). CommPath: an R package for inference and analysis of pathway-mediated cell-cell communication chain from single-cell transcriptomics. Comput. Struct. Biotechnol. J..

[28] Zhou Q. (2023). ChromLoops: a comprehensive database for specific protein-mediated chromatin loops in diverse organisms. Nucleic Acids Res..

[29] Maeser D., Gruener R.F., Huang R.S. (2021). oncoPredict: an R package for predicting in vivo or cancer patient drug response and biomarkers from cell line screening data. Brief Bioinform.

[30] Liu X. (2022). Let-7c-5p restrains cell growth and induces apoptosis of lung adenocarcinoma cells via targeting ESPL1. Mol. Biotechnol..

[31] Gui T., Yao C., Jia B., Shen K. (2021). Identification and analysis of genes associated with epithelial ovarian cancer by integrated bioinformatics methods. PLoS One.

[32] Wu F. (2022). Bioinformatics analysis of key genes and potential mechanism in cadmium-induced breast cancer progression. Environ. Sci. Pollut. Res. Int..

[33] Yang Q., Yu B., Sun J. (2020). TTK, CDC25A, and ESPL1 as prognostic biomarkers for endometrial cancer. BioMed Res. Int..

[34] Koedoot E. (2021). Splicing factors control triple-negative breast cancer cell mitosis through SUN2 interaction and sororin intron retention. J. Exp. Clin. Cancer Res..

[35] Guo G. (2013). Whole-genome and whole-exome sequencing of bladder cancer identifies frequent alterations in genes involved in sister chromatid cohesion and segregation. Nat. Genet..

[36] Park M. (2022). Breast cancer metastasis: mechanisms and therapeutic implications. Int. J. Mol. Sci..

[37] Wang Y., Yang Z. (2020). A Gleason score-related outcome model for human prostate cancer: a comprehensive study based on weighted gene co-expression network analysis. Cancer Cell Int..

[38] Zheng J., Zhang Y.W., Pan Z.F. (2021). [Dysregulation of mad2l1/camk2a/PTTG1 gene cluster maintains the stemness characteristics of uterine corpus endometrial carcinoma]. Zhongguo Yi Xue Ke Xue Yuan Xue Bao.

[39] Patel V.G., Oh W.K., Galsky M.D. (2020). Treatment of muscle-invasive and advanced bladder cancer in 2020. CA Cancer J Clin.

[40] Balar A.V. (2017). Atezolizumab as first-line treatment in cisplatin-ineligible patients with locally advanced and metastatic urothelial carcinoma: a single-arm, multicentre, phase 2 trial. Lancet.

[41] Bellmunt J. (2017). Pembrolizumab as second-line therapy for advanced urothelial carcinoma. N. Engl. J. Med..

[42] Feng Y., Jia B., Shen Z. (2022). Metformin and bladder cancer: drug repurposing as a potential tool for novel therapy: a review. Medicine (Baltim.).

[43] Jiménez-Guerrero R. (2018). Obatoclax and paclitaxel synergistically induce apoptosis and overcome paclitaxel resistance in urothelial cancer cells. Cancers.

[44] Coen J.J. (2019). Bladder preservation with twice-a-day radiation plus fluorouracil/cisplatin or once daily radiation plus gemcitabine for muscle-invasive bladder cancer: NRG/RTOG 0712-A randomized phase II trial. J. Clin. Oncol..

[45] Hu J. (2022). Neoadjuvant immunotherapy, chemotherapy, and combination therapy in muscle-invasive bladder cancer: a multi-center real-world retrospective study. Cell Rep Med.

[46] McElree I.M. (2023). Comparison of sequential intravesical gemcitabine and docetaxel vs Bacillus calmette-guérin for the treatment of patients with high-risk non-muscle-invasive bladder cancer. JAMA Netw. Open.

[47] Han M.A. (2021). Intravesical gemcitabine for non-muscle invasive bladder cancer. Cochrane Database Syst. Rev..

[48] Mohanta T.K., Mishra A.K., Al-Harrasi A. (2021). The 3D genome: from structure to function. Int. J. Mol. Sci..

[49] Hsieh T.S. (2022). Enhancer-promoter interactions and transcription are largely maintained upon acute loss of CTCF, cohesin, WAPL or YY1. Nat. Genet..

[50] Zuin J. (2022). Nonlinear control of transcription through enhancer-promoter interactions. Nature.

[51] Solomon D.A., Kim J.S., Waldman T. (2014). Cohesin gene mutations in tumorigenesis: from discovery to clinical significance. BMB Rep.

[52] Allegra A. (2022). Circular RNA as a novel biomarker for diagnosis and prognosis and potential therapeutic targets in multiple myeloma. Cancers.

[53] Kahraman M. (2018). MicroRNA in diagnosis and therapy monitoring of early-stage triple-negative breast cancer. Sci. Rep..

[54] Laengsri V. (2018). Cervical cancer markers: epigenetics and microRNAs. Lab. Med..

[55] Li C. (2020). MicroRNA-299-5p inhibits cell metastasis in breast cancer by directly targeting serine/threonine kinase 39. Oncol. Rep..

[56] Fateh A., Feizi M.A.H., Safaralizadeh R., Azarbarzin S. (2017). Importance of miR-299-5p in colorectal cancer. Ann. Gastroenterol..

[57] Wang Z. (2018). miRNA-299-5p regulates estrogen receptor alpha and inhibits migration and invasion of papillary thyroid cancer cell. Cancer Manag. Res..

[58] Zhang C.L. (2018). MiR-299-5p targets cell cycle to promote cell proliferation and progression of osteosarcoma. Eur. Rev. Med. Pharmacol. Sci..

[59] Azarbarzin S. (2016). The value of miR-299-5p in diagnosis and prognosis of intestinal-type gastric adenocarcinoma. Biochem. Genet..

